# The percutaneous permeation of a combination of 0.1% octenidine dihydrochloride and 2% 2-phenoxyethanol (octenisept^®^) through skin of different species *in vitro*

**DOI:** 10.1186/1746-6148-7-44

**Published:** 2011-08-11

**Authors:** Jessica Stahl, Michael Braun, Joerg Siebert, Manfred Kietzmann

**Affiliations:** 1 Department of Pharmacology, Toxicology and Pharmacy, University of Veterinary Medicine Hannover, Foundation, Buenteweg 17, 30559 Hannover, Germany; 2 Schülke & Mayr GmbH, Robert-Koch-Strasse 2, 22851 Norderstedt, Germany

## Abstract

**Background:**

A water based combination of 0.1% octenidine dihydrochloride and 2% 2 - phenoxyethanol is registered in many European countries as an antiseptic solution (octenisept^®^) for topical treatment with high antimicrobial activity for human use, but octenidine based products have not been registered for veterinary use yet. The aim of the present study was to investigate whether octenidine dihydrochloride or 2 -phenoxyethanol, the two main components of this disinfectant, permeate through animal skin *in vitro*. Therefore, permeation studies were conducted using Franz-type diffusion cells. 2 ml of the test compound were applied onto 1.77 cm^2 ^split skin of cats, dogs, cows and horses. To simulate wounded skin, cattle skin was treated with adhesive tapes 100 times, as well. Up to an incubation time of 28 hours samples of the acceptor chamber were taken and were analysed by UV-HPLC. Using the method of the external standard, the apparent permeability coefficient, the flux J_max_, and the recovery were calculated. Furthermore, the residues of both components in the skin samples were determined after completion of the diffusion experiment.

**Results:**

After 28 hours no octenidine dihydrochloride was found in the receptor chamber of intact skin samples, while 2.7% of the topical applied octenidine dihydrochloride permeated through barrier disrupted cattle skin. 2 - phenoxyethanol permeated through all skin samples with the highest permeability in equine, followed by bovine, canine to feline skin. Furthermore, both components were found in the *stratum corneum *and the dermis of all split skin samples with different amounts in the examined species.

**Conclusion:**

For 2-phenoxyethanol the systemic impact of the high absorption rate and a potential toxicological risk have to be investigated in further studies. Due to its low absorption rates through the skin, octenidine dihydrochloride is suitable for superficial skin treatment in the examined species.

## Background

Antiseptics are used to kill or inactivate microorganisms after topical administration on the surface of the body, in body cavities, and on surgically exposed tissues [1]. In recent years there has been a strongly growing interest in developing antiseptic drugs with best effectiveness against microbes and less potential of microbial resistance for both human and veterinary application. Thus, octenidine dihydrochloride (OCT), an alkanediylbis[pyridine] germicidal agent with structural similiarity to chlorhexidine and benzalkonium chloride, was designed as a new antiseptic drug with optimal antiseptic properties [1,2]: it is stable under exposition to light, temperature and in the pH range of 1.6-12.2, and its antiseptic effects are retained in the presence of albumin or mucin (no protein error) [1,3]. It is effective against yeasts and fungi [4,5], Gram-negative and -postive bacteria including actinomycetes, as well as plaque-forming bacteria [1,2,4,6-9]. Another good property of OCT is that low-level exposure of Methicillin-resistant Staphylococcus aureus (MRSA) does not develop resistance [10]. Today OCT it is used in human medicine in some licensed antiseptic products in several European countries (e.g. octenisept^® ^with 0.1% OCT and 2% 2-phenoxyethanol (PH)). Since OCT is an antiseptic drug with low cytotoxicity and high microbicidal effects in human medicine [5,11], it represents an auspicious candidate for antibacterial treatment in veterinary medicine. The antibacterial treatment of skin e.g. before surgery or wound treatment seems to be one main application of an antiseptic, but only few information is available about skin permeability and possible harmful body concentrations. To assess the suitability of OCT and PH for superficial disinfection of healthy and wounded skin in animals we investigated the *in vitro *percutaneous permeation of OCT and PH through intact skin of different species and barrier disrupted cow skin in diffusion cell experiments over 28 hours according to Stahl et al. (2010) [12].

## Methods

### Trial preparation

Octenisept^® ^was obtained from Schülke & Mayr GmbH, Norderstedt, Germany. It contained 0.1% OCT and 2% PH, together with an undisclosed amount of (3-amidopropyl cocoate)-dimethylammonium acetate, sodium D-gluconate, glyce-rol 85%, sodium chloride, sodium hydroxide, and purified water.

### Animals

All animals used in the present study died for reasons not related to this examination. Canine skin was obtained from animals that died in final experiments at the Institute of Parasitology, University of Veterinary Medicine, Foundation, Hannover. Equine and feline skin was excised from animals that suffered from uncurable diseases (University of Veterinary Medicine, Foundation, Hannover), while bovine udder skin was obtained from animals that died in slaughterhouse under legal requirements. Skin samples were obtained from the lateral abdominal skin, respectively, bovine udder and were stored for a maximum of 6 months at -20°C until use.

### Experimental setup

All studies were conducted according to the OECD-guideline 428 [13]. After gentle defrosting of the skin, the hair was clipped off (hair clipper: Wahl GmbH, Unterkirnach, Germany) and skin samples with a thickness of 500 μm were obtained using a dermatome (Zimmer, Freiburg, Germany). The skin samples were hydrated in phosphate buffered saline (PBS: 1.44 g disodium hydrogenphosphate, 8.0 g sodium chloride, 0.24 g potassium dihydrogenphosphate and 0.2 g potassium chloride; pH 7.4) for 30 minutes before use. Visual examinations were performed to study the skin integrity; the skin thickness was determined by a micrometer (Mitutoyo, Neuss, Germany 450-650 nm).

To induce barrier disruption in some bovine skin samples, the skin surface was treated with tape strips (Tesa, Beiersdorf, Hamburg, Germany) up to 100 times. The removal of all *stratum corneum *layers was ascertained by histological examination. Permeation experiments were conducted in Franz-type diffusion-cells (Permgear, Bethlehem, USA) with an average permeation area of 1.77 cm^2^. The receptor compartment contained 12 ml Soerensen phosphate buffer (9.2 g disodium hydrogenphosphate, 4.2 g sodium chloride and 2.0 g potassium dihydrogenphosphate, pH 7.4), which was stirred continously with a magnetic bar at 500 U/min. Since both components show low solubilities in Soerensen phosphate buffer (< 0.1 mg/ml), sink conditions have been assumed during the whole experiment. The donor compartment was filled with 2 ml octenisept^® ^(1.14 ml/cm^2^) and was covered with parafilm^®^. A water bath provided a constant temperature of 32°C in each diffusion cell. Samples were taken from the receptor compartment (800 μl) at each time point (1 h, 2 h, 3 h, 4 h, 5 h, 6 h, 22 h, 24 h, 26 h, 28 h) and were replaced with 800 μl Soerensen phosphate buffer.

High-performance liquid-chromatography (HPLC) was used to analyse the concentration of OCT and PH with the following HPLC conditions:

autosampler (508, Beckmann), column (LichroCART 125-4, 5 μm, 18 e, 10 cm, Merck), pre-column (LichroCART 4-A, 5 μm, Merck), heater (SpH 99, Spark Holland; 40°C), UV-VIS-detector (168, Beckmann).

The utilised mobile phase consisted of 80% Methanol and 20% McIlvaine-buffer (20.8 g Citric acid (anhydrous) and 0.4 g disodium hydrogenphosphate; pH 2.2) for OCT detection and 20% Methanol and 80% McIlvaine-buffer for PH detection. The detection wavelengths were 280 nm for OCT and 270 nm for PH, respectively. The flow rate was set up at 1.5 ml/minute with a pressure of ca. 20 mPa. 100 μl of each sample were injected into the HPLC.

The HPLC analysis was validated for both test substances in selectivity, linearity, accuracy, precision and stability. Selectivity was proven for each test substance in the chromatogram, and linearity was given between 600 and 10,000 ng/ml for OCT and between 400 and 10,000 ng/ml for PH. The accuracy and the precision were detected by analysis of low quality control standards (LQS) as well as high quality control standards (HQS) on three different days. Relative deviations < 20% (accuracy) and < 10% (precision) were accepted for LQS and < 15% for HQS (accuracy and precision). Stability was given for the whole time of the experiment. The limits of detection (LOD) were 243.1 ng/ml for OCT and 91.3 ng/ml for PH. The limits of quantification (LOQ) were 600 ng/ml for OCT and 400 ng/ml for PH. Quantitative analysis was based on comparison with the calibration curve, while the areas under the curve were analysed using the method of external standards. The calculation of the drug amount permeated through 1 cm^2 ^skin was calculated from the concentration in the receptor chamber according to Niedorf et al. (2008) [14] using an iterative algorithm to recalculate the flux and the transport, based on the actual concentration difference between the donor and acceptor compartments for short time intervals. The algorithm thus yields non-linear permeated mass per unit area *versus *time curves and calculates both the apparent permeability coefficient (P_app_) and the maximum flux (J_max_) after 28 hours.

The amount of OCT and PH in the *stratum corneum *and the dermis was determined after completion of the diffusion experiment. Therefore, the split skin samples were rinsed with cold water and the skin sheets were separated according to Kligman and Christophers (1963) [15]. The following extraction procedure was performed: Two ml PBS were added to each sample before homogenisation for 30 seconds. 20 μl H_2_SO_4 _(2 N) and 4 ml dichlormethane were added and the samples were shaken for 30 minutes. After centrifugation (10 minutes, 4°C, 10 × g) the lower phase comprising dichlormethane was transferred into another vial and was evaporated using compressed air. 0.5 ml eluent was used to dissolve the analytes. The recovery was approximately 90-95%.

## Results

**Figure 1:**
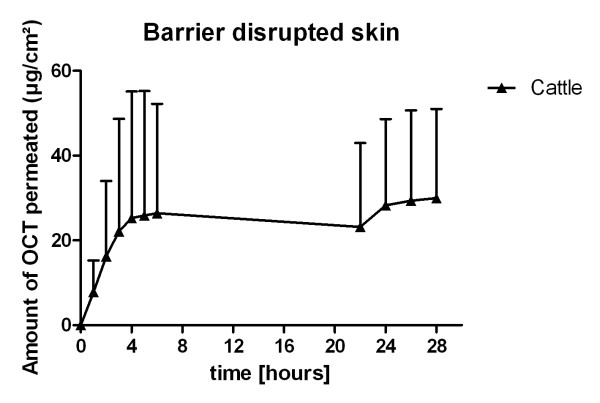
**Permeation rate of OCT**. Permeation rate curve of OCT obtained in diffusion experiments over 28 hours with barrier disrupted cattle skin (mean and standard deviation); no OCT was detected after 28 hours in histologically intact skin of cats, dogs, cattle and horses; (n = 6).

**Figure 2:**
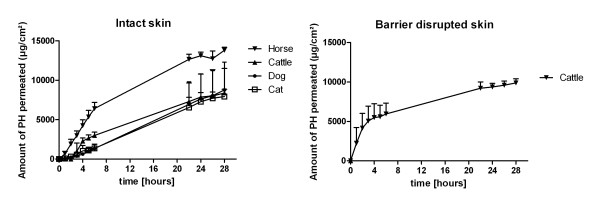
**Permeation rates of PH**. Permeation rate curve of PH obtained in diffusion experiments over 28 hours with histologically intact skin samples (left) and barrier disrupted cattle skin (right) (mean and standard deviation); (n = 6).

OCT was not detectable in the acceptor medium of the histologically intact skin of any examined species during the experiment (Figure 1), although OCT was found in the *stratum corneum *and the dermis of all examined split skin samples (Table 1). After 6 hours 2.34% and after 28 hours 2.64% of the applied OCT (2 mg) was determined in the acceptor fluid of barrier disrupted bovine skin (Figure 1). PH permeated through all skin samples (Figure 2), with the highest flux in equine skin followed by bovine udder skin, canine to feline skin. After barrier disruption in bovine udder skin a 1.8-fold higher flux J_max _was observed. After 6 hours the recoveries in the receptor compartment ranged from 5.9% (dog) to 28.4% (horse), while after 28 hours 35.1% (cat) to 61.1% (horse) were found. All data obtained in the diffusion experiments are shown in Table 2 and 3. The amount of PH storage in the skin layers is listed in Table 1.

**Table 1:** Amount of OCT and PH in animal skin layers

	Skin residue (μg/cm^2^)	Cattle		Cattle "barrier disrupted"		Dog		Cat		Horse	
		
		Mean	STD	Mean	STD	Mean	STD	Mean	STD	Mean	STD
	*Stratum corneum*	1.00	0.28	---	---	0.65	0.34	1.79	0.32	0.99	0.57
OCT	Dermis	3.36	1.81	5.10	1.15	2.79	0.94	4.67	1.61	2.58	1.01
	Total amount	4.37	1.84			3.44	0.99	6.46	1.76	3.57	1.37

	*Stratum corneum*	317.04	66.67	---	---	40.94	14.11	82.25	16.41	345.70	126.99
PH	Dermis	108.93	20.33	197.08	85.43	79.01	40.28	124.97	55.55	111.67	32.98
	Total amount	407.81	67.65			113.13	44.90	207.21	59.70	457.37	144.67

Amount of OCT and PH in the *stratum corneum *and the dermis of the split skin samples after 28 hours experiments (n = 4-6)

**Table 2:** Characteristics of PH permeability of histologically intact skin

Parameter	Cattle		Dog		Cat		Horse	
	
	Mean	STD	Mean	STD	Mean	STD	Mean	STD
J_max _(μg/cm^2^/h)	1231.04	162.65	392.06	159.09	580.87	379.56	1298.78	178.97
P_app _(cm/s)	1.71E-05	2.26E-06	5.45E-06	2.21E-06	8.07E-06	5.27E-06	1.80E-05	2.49E-06
Recovery 6 h (%)	13.25	1.86	5.88	2.28	6.00	2.35	28.36	3.48
Recovery 28 h (%)	37.01	2.18	38.80	15.68	35.09	15.79	61.10	1.64

Flux J_max_, apparent permeability coefficient P_app_, and recoveries after 6 and 28 hours of PH obtained in diffusion experiments with histologically intact split skin of different animals after 28 hours (n = 6) calculated from the maximum absorption according to Niedorf et al. (2008) [14]

**Table 3:** Characteristics of OCT and PH permeability of barrier disrupted skin

Parameter	OCT		PH	
	
	Mean	STD	Mean	STD
J_max _(μg/cm^2^/h)	9.43	9.24	2211.64	821.44
P_app _(cm/s)	2.62E-06	2.57E-06	3.07E-05	1.14E-05
Recovery 6 h (%)	2.34	2.29	26.27	6.14
Recovery 28 h (%)	2.65	1.87	43.67	2.38

Flux J_max_, apparent permeability coefficient P_app_, and recoveries after 6 and 28 hours of PH and OCT obtained in diffusion experiments with barrier disrupted split skin of cattle after 28 hours (n = 6) calculated from the maximum absorption according to Niedorf et al. (2008) [14]

## Discussion

This study provides information about percutaneous permeation of OCT and PH out of a licensed superficial disinfectant through skin of different animals *in vitro *and the distribution of both substances in the *stratum corneum *and the dermis after 28 hours diffusion experiment. Both components are used for topical surface treatment and are supposed to stay on the body surface or within the outmost skin layers to show best effectiveness against bacteria and other microorganisms. Due to its high molecular weight (550.9 g/mol) OCT should be retarded by the outermost skin layer, the *stratum corneum*. Thus, silimar to porcine skin [12] OCT is not detectable in the receptor fluid of histologically intact skin of cats, dogs, cows and horses out of a commonly available formulation containing 0.1% OCT and 2% PH after 28 hours under occlusive conditions, although it is present in the extracts from the *stratum corneum *and the dermal skin layers of all animals. The reason for OCT measurement in the dermis may be due to the fact that hair follicles arise from the dermis and represent storage compartments for several drugs [16]. Thus, it is likely that the amount of OCT measured in the dermal layers represents the capacity of substance storage in the hair follicles with considerable differences between the species depending on the amount and size of the hair orifices. Therefore, it can be assumed that topically applied OCT reaches the base of the hair follicles and affects microorganisms within this hideaway. To study the effect of *stratum corneum *disruption in damaged skin the tape stripping procedure, an experimental setup suitable for skin with low hair density [17-20], was used in bovine udder skin. But even after barrier disruption, only low amounts of OCT were found in the receptor compartment of bovine skin (2.64% after 28 hours), which has also been demonstrated for barrier disrupted porcine skin [12]. It is likely that OCT which reaches the dermis in barrier disrupted skin samples attaches to dermal surface structures, since OCT contains four tertiary amino groups, two of which are protonated, and shows a high affinity to negatively charged surfaces [1]. Thus, the amount of OCT found in the receptor compartment of barrier disrupted skin samples is quite moderate, despite *stratum corneum *removal.

PH, the other constituent of the used disinfectant combination, was found in all receptor samples with recoveries of 5.9% (dog) to 28.4% (horse) after 6 hours to 35.1% (cat) to 61.1% (horse) after 28 hours, whereas porcine skin, which is comparable to human skin regarding permeability, exhibits recoveries of only 1.4% after 6 hours to 11.3% after 28 hours [12]. Interspecies differences in the permeation rates may be due to skin characteristics like morphology and lipid composition or the interaction of the formulation with the different skin types [21-23]. Similar to Bronaugh et al. (1986) [24] permeation differences caused by different storage times could be ruled out for PH (data not shown), although it has been demonstrated that freezing increases skin permeability e.g. for hydrocortisone through canine full thickness skin [25]. It is likely, that an enhancing effect of freezing on PH permeability was not detectable, since PH permeates relatively quickly through intact animal skin out of the examined formulation and significant increases in permeability may thus be harder to be found. High *in vitro *permeability rates of PH have also been observed for rat and human skin. Roper et al. (1997) showed that depending on the receptor fluid used in the diffusion cell experiment 43-64% PH permeated within 28 hours, while occlusive dressing led to 99% permeability [26]. It has also been described to be readily absorbed by newborn's skin with extensive oxidative metabolisation to 2-phenoxyacetic acid [27]. However, first-pass metabolism of PH was not detectable in our study with skin samples, all of which stored in the freezer, or during percutaneous permeation through viable rat and human skin *in vitro *[20].

The fact that skin damage caused by tape stripping procedure resulted in a moderate 1.8-fold higher flux of PH through bovine skin in comparison to a 10.2-fold higher flux through porcine skin [12] leads to conclusion that the *stratum corneum *in porcine skin maintains a stronger permeation barrier than in bovine udder skin, which has also been demonstrated for several non-steroidal anti-inflammatory drugs [28], all of which exhibited an inverse correlation of permeation with *stratum corneum *thickness. Therefore, porcine skin with considerable retention of PH by the *stratum corneum *is stronger affected by removal of the horny layer than bovine skin, which already exhibits high permeation rates in physiologically intact skin.

## Conclusion

After topical treatment of healthy animal skin with a combination of OCT and PH just PH permeates through the skin, while OCT permeates the skin only in small amounts after barrier damage. The systemic impact of the high absorption rate of PH and a potential toxicological risk have to be investigated in further studies or should lead to conclusion to avoid PH in disinfectants used for topical treatment of the investigated species. In contrast, the low absorption rates of OCT demonstrate its suitability for topical disinfection.

Taking into account the good efficacy of OCT against a wide range of micro-organisms, it represents a promising antibacterial agent for superficial skin treatment in animals.

## Abbreviations

OCT: Octenidine dihydrochloride; PH: 2-Phenoxyethanol; MRSA: Methicillin-resistant Staphylococcus aureus; LOD: Limit of detection; LOQ: Limit of quantification; HPLC: High-performance liquid-chromatography; HQS: High quality control standards; LQS: Low quality control standards.

## Authors' contributions

JS carried out the permeation studies and drafted the manuscript. All authors participated in the design of the study and read and approved the final manuscript.

## Authors information

MB and JS are employees of Schülke & Mayr GmbH, Norderstedt, Germany. The author(s) declare that they have no competing interests.
